# FEDERAL POLICY SUPPORTS AND GAPS IN ADDRESSING RACIAL-ETHNIC HEALTH DISPARITIES IN U.S. LONG-TERM CARE FACILITIES

**DOI:** 10.1093/geroni/igz038.576

**Published:** 2019-11-08

**Authors:** Rebecca L Mauldin, Kathy Lee, Antwan Williams

**Affiliations:** University of Texas at Arlinton, Arlington, Texas, United States

## Abstract

Older adults from racial and ethnic minority groups face health inequities in long-term care facilities such as nursing homes and assisted living facilities just as they do in the United States as a whole. In spite of federal policy to support minority health and ensure the well-being of long-term care facility residents, disparities persist in residents’ quality of care and quality of life. This poster presents current federal policy in the United States to reduce racial and ethnic health disparities and to support long-term care facility residents’ health and well-being. It includes legislation enacted by the Patient Protection and Affordable Care Act of 2010 (ACA), regulations of the U.S. Department of Health and Human Services (DHHS) for health care facilities receiving Medicare or Medicare funds, and policies of the Long-term Care Ombudsman Program. Recommendations to address threats to or gaps in these policies include monitoring congressional efforts to revise portions of the ACA, revising DHHS requirements for long-term care facilities staff training and oversight, and amending requirements for the Long-term Care Ombudsman Program to mandate collection, analysis, and reporting of resident complaint data by race and ethnicity.

